# An adaptive switching filter based on approximated variance for detection of impulse noise from color images

**DOI:** 10.1186/s40064-016-3644-9

**Published:** 2016-11-14

**Authors:** K. Pritamdas, Kh. Manglem Singh, L. Lolitkumar Singh

**Affiliations:** 1 Electronics and Communication Engineering (ECE), NIT Manipur, Takyelpat, Imphal, 795001 India; 2 CSE, NIT Manipur, Takyelpat, Imphal, India; 3 ECE, Mizoram University, Aizawl, India

**Keywords:** Cumulative, Exponential, Impulse noise, Variance, Vector

## Abstract

A new adaptive switching algorithm is presented where two adaptive filters are switched correspondingly for lower and higher noise ratio of the image. An adaptive center weighted vector median filter is used for the lower noise ratio whereas for higher noise ratio the noisy pixels are detected based on the comparison of the difference between the mean of the vector pixels in the window and the approximated variance of the vector pixels in the window. Then the window comprising the detected noisy pixel is further considered where the pixels are given exponential weights according to their similarity to the other neighboring pixels, spatially and radio metrically. The noisy pixels are then replaced by the weighted average of the pixels within the window. The filter is able to preserve higher signal content in the higher noise ratio as compared to other robust filters in comparison. With a little high in computational complexity, this technique performs well both in lower and higher noise ratios. Simulation results on various RGB images show that the proposed algorithm outperforms many other existing nonlinear filters in terms of preservation of edges and fine details.

## Background

Filtering is one of the most essential steps in the applications of image processing. An image must contain the required data to show the correct information before it is used for any image processing application. But images are usually corrupted with unwanted information that causes hindrance to an efficient image processing operations. These unwanted information which are termed as noise must be removed properly from the image as a preprocessing step. Additive random noise (Gaussian noise) and salt and pepper noise are some of the most common noises found in digital image. Impulse noise which may be fixed valued noise (FVN) or random valued noise (RVN) is one of the most naturally occurring noises in digital images and it is induced in the image during image acquisition by faulty sensors or during transmission through communication channels. Noise removal techniques depends on the type of noises degrading the image and also largely on the percentage of noise corrupting the image. A number of robust filters have been proposed in literature for filtering the color images corrupted with impulse noise. Non-linear filters which actually work in spatial domain suit well for impulse noise removal from color images (Celebi et al. 2007).

Initial approach like the marginal median filter (MMF) treats the color image channel wise in a scalar form which often leads to color artifacts (Pitas 1990). The nonlinear filters like the vector median filter (VMF) (Astola et al. 1990) and the basic vector directional filter (BVDF) (Trahanias and Venetsanopoulos 1992) which consider the color pixels as vectors and work on the concept of order-static filters, are very efficient for the impulse noise removal of color images. The VMF forms a sorted array of the cumulative distance of intensity value of the vector pixels from the surrounding pixels in the window, and then the corresponding vector pixel which gives the least value of cumulative distance in the sorted array is substituted as the vector median instead of the center pixel. And in case of VDF, the sorted array is of the cumulative angular distance of the vector pixels from the surrounding vectors in the window. Thus the output of the VDF is the vector pixel that corresponds to the least value of cumulative angular distance. The directional-distance filter (DDF) (Karakos and Trahanias 1995) combines the *μ* magnitude part from the VMF and the (1 − *μ*) angular part from the VDF in calculating the cumulative distances from a vector pixel to the other in the filtering window. The center weighted VMF (CWVMF) (Smolka et al. 2012a), center weighted VDF (CWVDF) and the center weighted DDF (CWDDF) highlight the center pixel by assigning more weight. These filters have a tendency to preserve the center pixel in the filtering window which reduces the efficiency in higher noise ratio. These are the popular filters where the filtering is done uniformly across the pixels without using an actual noise detection algorithm. These filters tend to modify the uncorrupted pixels which result in blurring of the edges and loss of fine details of the image.

To overcome this particular issue noise detection schemes are introduced in the rank order static filters (Lukac 2004, Lukar and Smolka 2003), that check whether the center pixel is noisy or not. Then the noisy pixel is replaced by the output of a vector filter otherwise it is left unaltered. The adaptive CWVMF (ACWVMF) (Lukac and Smolka 2003), adaptive CWVDF (ACWVDF) (Lukac 2004) and adaptive CWDDF (ACWDDF) replace the center pixel by the output of VMF, VDF and DDF respectively, if the difference between the center pixel and the corresponding center-weighted vector median is greater than a user specified threshold. A weight in the range of 0–1 is given to cumulative distance of the center pixel, for getting the vector median of the modified CWVMF (MCWVMF).

In the peer group filter (PGF) the pixels in the window is sorted according to their differences from the center pixel, then a peer group of $m = {{\left( {\sqrt n + 1} \right)} \mathord{\left/ {\vphantom {{\left( {\sqrt n + 1} \right)} 2}} \right. \kern-0pt} 2}$ is selected from the sorted array, where *n* is the number of vector pixels in the window. If the difference between any two pixels from the peer group is greater than user-specified threshold, then the center pixel is replaced with the output of VMF. And if the difference of the individual pixel, in the peer group *m*, from the center pixel is less than a user specified threshold, then the center pixel is replaced with the VMF, which results in the fast PGF (FPGF) (Kenny et al. 2001; Smolka and Chydzinski 2005; Malinski and Smolka 2015). The adaptive VMF (AVMF) and adaptive VDF (AVDF) replace the center pixel with the output of VMF and VDF respectively if their respective cumulative distance is greater than a user specified threshold $\partial$ and *T* (Lukac 2002, 2003).

The entropy VMF (EVMF), entropy VDF (EVDF) and entropy DDF (EDDF) are the group of entropy vector filters which replace the center pixel with the output of VMF, VDF and DDF respectively, if the local contrast entropy of the center pixel is greater than its local contrast entropic threshold multiplied by a weighting factor *υ* (Lukac et al. 2003).

The Neuvo VMF (NVMF) (Sun and Neuvo 1994) is that kind of vector filter where the center pixel is considered to be noisy if its difference from the vector median is bigger than a predefined threshold. For the rank-conditioned VMF (RCVMF), rank-conditioned VDF (RCVDF) and rank-conditioned DDF (RCDDF), with respect to ascending order of the respective cumulative distances, a subset of the vector pixels excluding the minimum and maximum values is formed (Singh and Bora 2004). If the center pixel is not in the ordered subset, then it is replaced by the output of VMF, VDF and DDF respectively. If *l* is the number of pixels in the ordered set, then position of the noisy center pixel will be outside the range of *l*. In the rank-conditioned and threshold VMF (RCTVMF), rank-conditioned and threshold VDF (RCTVDF) and rank-conditioned and threshold DDF (RCTDDF) (Singh and Bora 2004; Smolka et al. 2012b), the center pixel is considered as noisy if it does not belong in the trimmed set and its difference from the respective vector median is greater than a pre-defined threshold.

The group of fuzzy weighted filters namely the fuzzy vector median filter (FVMF), fuzzy vector directional filter (FVDF) and fuzzy directional distance filter (FDDF) weight the pixels in the filtering window according to their respective cumulative distance before replacing the center pixel with the average of the weighted pixels. These filters use certain parameters *α* and *β* to adjust amount of fuzziness in weighting the pixels (Plataniostis et al. 1996, 1999). The fuzzy ordered vector filter (FOVMF) picks up the first *l* elements of the ordered set of cumulative distances, and then assigns the weight only to the corresponding pixels.

In the signal dependent rank-ordered mean (SDM), if the difference between the center pixel and the first four pixels of the rank-ordered set of pixels is greater than four separate thresholds *T*_*a*_, *T*_*b*_, *T*_*c*_ and *T*_*d*_ respectively, Moore et al. (1999) then the center pixel is replaced with the output of VMF. The robust switching vector median filter (RSVMF) proposed by Celebi and Aslandogan considers the noisy pixel to be a noisy pixel if the cumulative distance with respect to the center pixel is greater than a predefined percentage *β* of the cumulative distance associated with the median (Celebi and Aslandogan 2008).

The non-causal linear predictor based filters use the concept of linear prediction (Singh 2012; Singh and Bora 2003) for estimating the center pixel as a weighted combination of past and future vector pixels with respect to the center pixel. It is based on the fact that there exists a strong correlation among the neighborhood pixels of a window centered at a vector pixel.

**Fig. 1:**
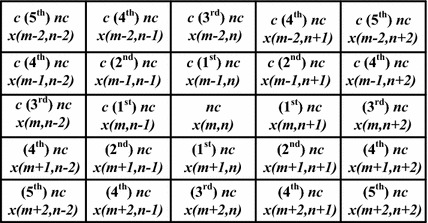
Block of causal and non causal regions showing different orders

The non-causal vector median filter (NCVMF) (Singh and Bora 2014) first filters the image using VMF, then estimates the center pixel using constrained intra-channel linear predictor (Hu et al. 1997) by considering the eight second order pixels (see Fig. 1) (David and Ramamurthi 1991; Asif and Moura 1996; Asif 2004). Then if the difference between the predicted pixel and the center pixel is greater than or equal to a user-specified threshold, the center pixel is replaced by the output of VMF, VDF or DDF. In the rank conditioned non-causal vector median filter (NCRVMF), the value of index *p*, which corresponds to the least valued element from the sorted array of cumulative distance of the vector pixels in the window, is compared with a threshold, which is calculated based on the standard deviations of the separate three channels of the image. If the value of *k* is greater than that of the threshold *T*, then the center pixel is predicted considering the 2nd order non-causal regions. The non causal vector median filter 2nd order non causal (NCVMF_2nc) considers only the upper four non causal pixels out of the eight, from the 2nd order region excluding the center pixel. The non causal vector median filter 1st order non causal (NCVMF_1nc) considers the four 1st order pixels from the non causal region. The non causal vector median filter 1st order causal (NCVMF_1c) predicts the center pixel using the two pixels of the 1st order causal region.

The adaptive vector marginal median filter (AVMMF) compares the cumulative distance of the center pixel with that of the sorted array of the cumulative distance of all the pixels in the window, so that center pixel lies in the *l* index in the sorted array (Morillas et al. 2011). And if *l* is greater than the index defining the center of the window, then the center pixel is replaced by the median of the vector medians for *n* = 1, 2, …, *k*, where *n* is the index of the sorted array of cumulative distances of the pixels in the window.

The vector sigma filters (Lukac et al. 2006) which are based on approximated variance of the vector pixels in the window are grouped into adaptive and non-adaptive. The multivariate variance is calculated either based on the vector mean or the lowest ranked vector, vector median. In the non-adaptive group of vector sigma filters the center pixel is replaced with the output of VMF, VDF and DDF, if the cumulative distance of the center pixel is greater than a threshold, calculated based on the cumulative distance of the mean vector or the vector median and a parameter *μ*. This results to the corresponding non-adaptive sigma vector filters as SVMF_MEAN, SVMF_RANK, SVDF_MEAN, SVDF_RANK, SDDF_MEAN and SDDF_RANK respectively. Then the groups of adaptive sigma vector filters are ASVMF_MEAN, ASVMF_RANK, ASVDF_MEAN, ASVDF_RANK, SDDF_MEAN and ASDDF_RANK correspondingly. Here the center pixel is replaced with output of VMF, VDF and DDF respectively if the difference between the center pixel and the mean vector or the vector median is greater than the product of the corresponding approximated variance and a weighting parameter *μ*, where the approximated variance is calculated based on the cumulative distance of the vector pixels in the window from that of the mean vector.

Many robust filters for impulse noise removal have been proposed in the literature but most of them which work efficiently in the lower noise ratio, lack considerably in the higher noise ratio. Filters like CWVMF, CWVDF, CWDDF, RCTVDF, RCTVDF, RCTDDF etc. which perform very well in the lowest noise ratio group, are not efficient in the higher noise ratio group. On the other hand filters like ASVMF_MEAN, ASDDF_MEAN and EVMF work quite well both in lowest and highest noise ratio group.

In this work, a simple algorithm checks the impulse noise ratio in the image prior to the implementation of the noise detection algorithms, depending on which two filters are switched accordingly from lower noise ratio to higher and vice versa. In the higher nose ratio, the noisy pixels are identified with the help of noise detection algorithm, based on how much the center pixel is different from the mean vector of the neighboring vectors in the widow (Lukac et al. 2006). Then the detected noisy pixels are replaced with the output of modified exponentially weighted mean filter (Celebi et al. 2007) based on the concept of bilateral filter (Tomashi and Manduchi 1998; Daniel John 2013; Kaur et al. 2015), whereas the uncorrupted pixels are kept untouched.

“Proposed method” section describes the proposed method. Noise model performance measuring parameters are described briefly in the next “Filter evaluation” section and finally the details are summarized and concluded in “Conclusions” section.

## Proposed method

**Fig. 2:**
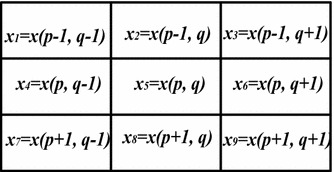
A 3 × 3 window of color vectors with their coordinates

Let a color image *X* of size *P* × *Q* be represented by a 2-D array of 3 component vectors represented as,

1 $$ \varvec{x}(p,q) = \left[ {x^{R} (p,q),x^{G} (p,q),x^{B} (p,q)} \right]\, $$

where $p = 1,2,3 \ldots P$ and $q = 1,2,3 \ldots Q$ represents the row and column indices. *R*, *G* and *B* represent the Red, Green and Blue components of the vector pixel ***x***(*p*,*q*). As usual in the basic filtering approach, a window *W* of odd size *m* × *n* centered at ***x***(*p*,*q*) is considered (see Fig. 2). Considering the fact that impulse noise is distributed randomly which results in the variation of noise content in different parts of the image, before checking whether the center pixel is noisy or not, a localized impulse noise probability *P*_*r*_, in the window comprising the center pixel is calculated as,

2 $$ p_{r} = N_{imp} /{m \times n}$$

where *N*_*imp*_ is the number of 0 s and 255 s in the window. If $T_{L} \le p_{r} \le \,T_{H}$, then the center pixel is replaced with the output of the ACWVMF, otherwise it is replaced with the output of the modified ASVMF_MEAN, MASVMF_MEAN, to give the overall proposed noise percentage based switching filter, NPSF as shown below

3 $$ \varvec{x}_{NPSF} = \,\,\left\{ \begin{array}{ll} \varvec{x}_{ACWVMF} &\quad if\,\,T_{L} \le p_{r} \le T_{H} \hfill \\ \varvec{x}_{MASVMF\_MEAN} &\quad otherwise \, \hfill \\ \end{array} \right. $$

*T*_*L*_ and *T*_*H*_ are the lower and higher threshold value of *p*_*r*_. *x*_*NPSF*_ is the output of NPSF and *x*_*ACWMF*_ is the output of ACWVMF which is defined as

4 $$ \varvec{x}_{ACWVMF} = \left\{ \begin{array}{ll} \varvec{x}_{VMF}&\quad if\,\,\sum\nolimits_{u = \nu }^{\nu + 2} {\left\| {\varvec{x}_{{CWVMF^{u} }} - \varvec{x}(p,q)} \right\| > \,T,} \hfill \\ \varvec{x}(p,q)&\quad otherwise \hfill \\ \end{array} \right.\quad \nu \in \,[1,\,c - 1]\, $$

where $c = {{((m \times n) + 1)} \mathord{\left/ {\vphantom {{((m \times n) + 1)} 2}} \right. \kern-0pt} 2}$, *T* is the threshold, with which the difference of the center pixel from the output of CWVMF is compared with and *x*_*CWVMF*_ is the output of CWVMF which is given as

$$ \varvec{x}_{{CWVMF^{u} }} = \,\arg \min_{{x_{i} \in \,W}} \,\left( {\sum\limits_{j = 1}^{m \times n} {w_{j} } (u).\,\left\| {\varvec{x}_{i} - \varvec{x}_{j} } \right\|} \right) $$

and

5 $$ w_{j} (u) = \left\{ {\begin{array}{*{20}l} {m \times n - 2u + 2} \hfill &\quad {for\,\,j = \,c} \hfill \\ 1 \hfill &\quad {otherwise} \hfill \\ \end{array} } \right.,\quad \,u \in \,[1,\,c]\, $$

represents the weight to be given to the center pixel.

ACWVMF is chosen for the window with less number of impulse noises, like if at the most one number of 0 or 255 is present. It is a very robust filter which is very efficient and flexible in removing impulse noise, which actually allows one to design an optimal filter for a particular domain by adjusting the weights assigned to the center pixel. The weights are estimated using an optimization procedure in Eq. 5 by using a number of training images (Celebi et al. 2007). *u* is used as the smoothing parameter such that if *u* = 1 the ACWVMF becomes an identity filter and so no smoothing will be performed and if the value of *u* increases from 1 to 5, the smoothing potential of the filter increases. When *u* reaches the value of *c*, lastly the ACWVMF becomes the VMF where the maximum amount of smoothing is done. In this paper three values of *u* is chosen from *v* to *v* + 2, for which three respective CWVMF outputs are determined to be subtracted from their respective center pixel as seen in Eq. 4. And if the sum of the three differences is greater than the threshold *T* then the center pixel is replaced with the output of VMF. In the ACWVMF the center pixel is highlighted or given more importance without a prior knowledge of whether it is a noisy pixel or not, by assigning a variable weight by Eq. 5, that makes the ACWVMF more suitable to be considered for windows which contain at the most one 0 or 255. The ACWVMF has a tendency to preserve the center pixel that makes it more suitable for lower noised region of the image.

**Fig. 3:**
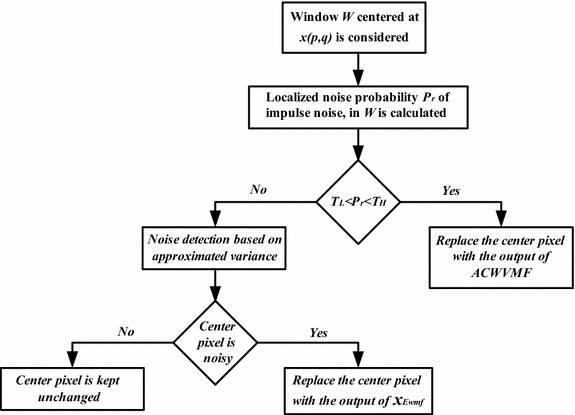
Flowchart of the proposed algorithm

And for the window with two or more number of 0 s and 255 s, a detection algorithm based on ASVMF_MEAN is used where the noisy pixel is detected based on the comparison of the center pixel with that of an approximated variance of the vector pixels in the filtering window (see Fig. 3). Depending on the fact that the impulse noise usually has very high or very low pixel value as compared to the surrounding pixels, the variance is calculated based on the mean vector of the pixels in the window, where mean is considered as one of the most probable values other than the vector median, for substituting the noisy pixel. Once the noisy pixels are detected, they will be replaced by the output of an exponentially weighted filter. The complete algorithm of noise detection using the approximated variance and replacement of the center pixel by the output of the exponentially weighted filter, defines the MASVMF_MEAN.

The variance *σ* ^2^, which is to be compared with, is calculated as,

6 $$ \sigma^{2} = \frac{1}{m \times n}\sum\limits_{i = 1}^{m \times n} {\left\| {x_{i} - \,x_{mean} } \right\|}^{2} $$

where,

7 $$ \,\left\| {\varvec{x}_{i} - x_{mean} \,} \right\|^{2} = \,\,\left| {\varvec{x}_{i}^{R} - x_{mean}^{R} } \right|^{2} + \left| {\varvec{x}_{i}^{G} - x_{mean}^{G} } \right|^{2} + \left| {\varvec{x}_{i}^{B} - x_{mean}^{B} } \right|^{2} $$

***x***_*mean*_ is the mean of the vector pixels in the window *W*. For checking the pixels to be noisy or not, the difference of the center pixel, ***x***(*p*,*q*) from the mean, ***x***_*mean*_ is compared with the approximated variance, *σ* ^2^ multiplied by a weighting factor $\partial$, to give $\partial \times \sigma^{\,2}$ and if $\left\| {x(p,q) - x_{mean} } \right\| \ge \partial \times \sigma^{\,2}$, then the centered pixel ***x***(*p*,*q*) is considered to be a noisy pixel.

This particular detection algorithm based on ASVMF_MEAN is suitable for higher noise region of the image because the approximated variance is calculated based on the cumulative difference of the vector pixels from the mean, which means that all the pixels whether impulse noise or not are considered and are given similar importance. And the vector pixel is treated as impulse noise if it is having a bigger difference from the mean, as compared to average of the difference of vector pixels from the mean as seen in Eqs. 6 and 7. Then the window where the center pixel is detected as noisy pixel is further considered for further classification of the vector pixels among themselves with respect to their individual importance in the window. This further classification is helpful for window with more number of impulse noises where more number of 0 s and 255 s are present as it helps in more efficient detection of noisy pixel. The classification and the corresponding assignment of weights to the respective vector pixel according to their importance in the window is given by the equation

8 $$ w_{i} = e^{{ - \,\,\left( {\frac{{d(i)^{\alpha } + l(i)^{\eta } }}{\beta }} \right)}} \quad for\,\,i = \,1,2,3, \ldots ,\,m \times \,n\, $$

where

9 $$ d(i) = \sum\limits_{j = 1}^{m \times n} {s\left( {\varvec{x}_{i} ,\varvec{x}_{j} } \right)} $$

10 $$ s(\varvec{x}_{i} ,\varvec{x}_{j} ) = \left\| {\varvec{x}_{i} - \varvec{x}_{j} } \right\|\, $$

11 $$ l(i) = \sum\limits_{j = 1}^{m\, \times \,n} {c\left( {\varvec{x}_{i} ,\varvec{x}_{j} } \right)} \, $$

And

12 $$ c(\varvec{x}_{i} ,\varvec{x}_{j} ) = \left\| {i - j} \right\|^{2} $$

Then the noisy pixel is replaced with the output of the exponentially weighted mean filter given as

13 $$ \varvec{x}_{Ewmf} = \frac{{\sum\nolimits_{i = 1}^{m \times n} {w_{i} \varvec{x}_{i} } }}{{\sum\nolimits_{i = 1}^{m \times n} {w_{i} } }} $$

Abiding by the normalization procedure, two constraints are necessary to make it sure that the output of *Ewmf* is an unbiased one, namely: (a) each weight $y_{i} = \frac{{w_{i} \,}}{{\sum\nolimits_{i = 1}^{m\, \times \,n} {w_{i} } }}$ for a respective vector pixel ***x***_i_ is a positive number, *y*_*i*_ ≥ 0 and (b) $\sum\nolimits_{i}^{m\,\, \times \,n} {y_{i} = 1}$. The parameters *α*, *η* and *β* are used to tune the amount of weight to be given to the pixels. To obey the above constraints so that the output is unbiased, it is made that $\eta = 1 - \alpha$ where $0\, < \,\,\alpha \,\, < \,\,1$ since the values of *d*(i) and *l*(i) will be in the range of *k* where $0\, \le k\,\, < \infty$ excluding $0\, < k\, < 1$. And *β* is the value of *α* at which the weighting function *w*_*i*_ takes the maximum derivative. The value of *η* = 1 − *α* also makes it sure that the two components in Eq. 8 are dependent and correlated to each other according to the noise probability of the sliding window. The first component described by Eqs. 9 and 10, depends on the cumulative distance of intensity difference of each pixel from its surrounding neighboring pixels. If the value of *d*(*i*) for a particular pixel *x*_*i*_ is larger, than it means that the intensity difference of the particular pixel from its surrounding is very large and has more tendencies to be an impulse noisy pixel. Therefore less weight is given to the pixel and vice versa. Whereas the second component as described by Eqs. 11 and 12, depends on the cumulative distance of coordinate or position difference of the pixel from its surrounding pixels. It is based on the fact that a pixel which is far away from the center pixel spatially is of less importance. Therefore a pixel ***x***_i_ with a large value of *l*(*i*) is far away from the center pixel ***x***(*p*,*q*) and is of less resemblance or importance, to be assigned with less weight. And it can be also seen that the parameters *α* and *η* are inversely proportional to the weight *w*_*i*_.

Further for a particular value of *d*(i) and *l*(i), the amount of weight assigned is again controlled by the respective values of *α* and *η*. If any of *α* or *η* is having a value of 1 then the other will be 0 that avoids the condition for both the components to be considered simultaneously. The condition that $\eta = 1 - \alpha$ makes it achievable that, for the window with a comparatively lesser number of 0 s and 255 s in the region of $p_{r} \, > \,T_{H}$ and with a lesser value of *d*(*i*), more weight is allowed to be assigned to the pixel by the first component of Eq. 8 with a lesser value of *α*, thus highlighting the first component, that subsequently makes the second component which is made of *l*(i) to be less important with a comparatively higher value of $\eta = 1 - \alpha$. Whereas in the window with more number of 0 s and 255 s which usually has a higher value of *d*(*i*), less weight is made to be assigned by the first component with higher value of *α* and thus the second component is given more importance with a comparatively lesser value of $\eta = 1 - \alpha$. This fact is supported by the results in Tables 1 and 2 where the values of NCD, PSNR, MAE and TIME are listed at different noise ratios and at different values of *α* and *η* for fixed valued and random valued noise correspondingly. And the values of *α* and *η* at which the value of PSNR and other measuring parameters attain their maximum values is highlighted. The selection of values for the parameters is further discussed in part C: Parameter selection, of section III.

**Table 1:** Various measuring criteria and their values at different values of *α* and *η* at different noise ratio, for the proposed filter for FVN

Values of *α* and *η*	FVN				FVN				FVN				FVN			
	.10				.20				.80				.90			
	NCD	PSNR	MAE	TIME	NCD	PSNR	MAE	TIME	NCD	PSNR	MAE	TIME	NCD	PSNR	MAE	TIME
Lower values of *α*																
0.5, 0.5	0.0043	37.242	0.669	5.450	0.005	35.511	1.139	7.260	0.017	27.312	5.61	15.44	0.0220	24.946	7.90	16.44
0.4, 0.6	0.0040	37.315	0.668	5.563	0.005	35.541	1.148	7.475	0.020	26.335	6.66	16.37	0.0221	23.928	9.62	17.25
0.3, 0.7	0.0040	37.246	0.689	5.574	0.005	35.074	1.242	7.420	0.024	23.967	9.83	16.17	0.0302	21.868	13.6	17.34
Higher values of *α*																
0.6, 0.4	0.0058	37.169	0.670	5.506	0.005	35.421	1.145	7.458	0.014	27.568	5.28	16.23	0.0218	25.893	6.80	17.27
0.7, 0.3	0.0058	37.204	0.669	5.559	0.005	35.577	1.137	7.379	0.018	27.866	5.15	16.28	0.0201	25.931	6.65	17.35
0.8, 0.2	0.0054	37.222	0.674	5.545	0.005	35.291	1.163	7.483	0.017	27.508	5.25	16.34	0.0211	25.851	6.66	17.37

**Table 2:** Various measuring criteria and their values at different values of *α* and *η* at different noise ratio, for the proposed filter for RVN

Values of *α* and *η*	RVN				RVN				RVN				RVN			
	.10				.20				.80				.90			
	NCD	PSNR	MAE	TIME	NCD	PSNR	MAE	TIME	NCD	PSNR	MAE	TIME	NCD	PSNR	MAE	TIME
Lower values of *α*																
0.5, 0.5	0.0017	36.928	0.666	3.90	0.003	35.152	1.134	4.504	0.015	27.618	5.32	8.72	0.0219	25.981	6.84	9.52
0.4, 0.6	0.0016	37.063	0.655	3.95	0.003	35.161	1.147	4.560	0.018	27.239	5.67	9.07	0.0227	25.611	7.39	9.92
0.3, 0.7	0.0017	37.056	0.667	4.02	0.003	34.972	1.171	4.711	0.019	25.967	7.03	9.08	0.0253	24.105	9.48	9.90
Higher values of *α*																
0.6, 0.4	0.0017	37.023	0.658	4.17	0.002	35.102	1.135	4.616	0.016	27.748	5.28	9.06	0.0212	26.237	6.65	9.85
0.7, 0.3	0.0018	37.017	0.660	4.04	0.003	35.042	1.143	4.623	0.017	27.707	5.23	9.06	0.0204	26.134	6.68	9.92
0.8, 0.2	0.0019	36.899	0.668	3.99	0.005	35.092	1.144	4.640	0.017	27.662	5.25	9.10	0.0215	25.980	6.73	9.92

## Filter evaluation

**Fig. 4:**
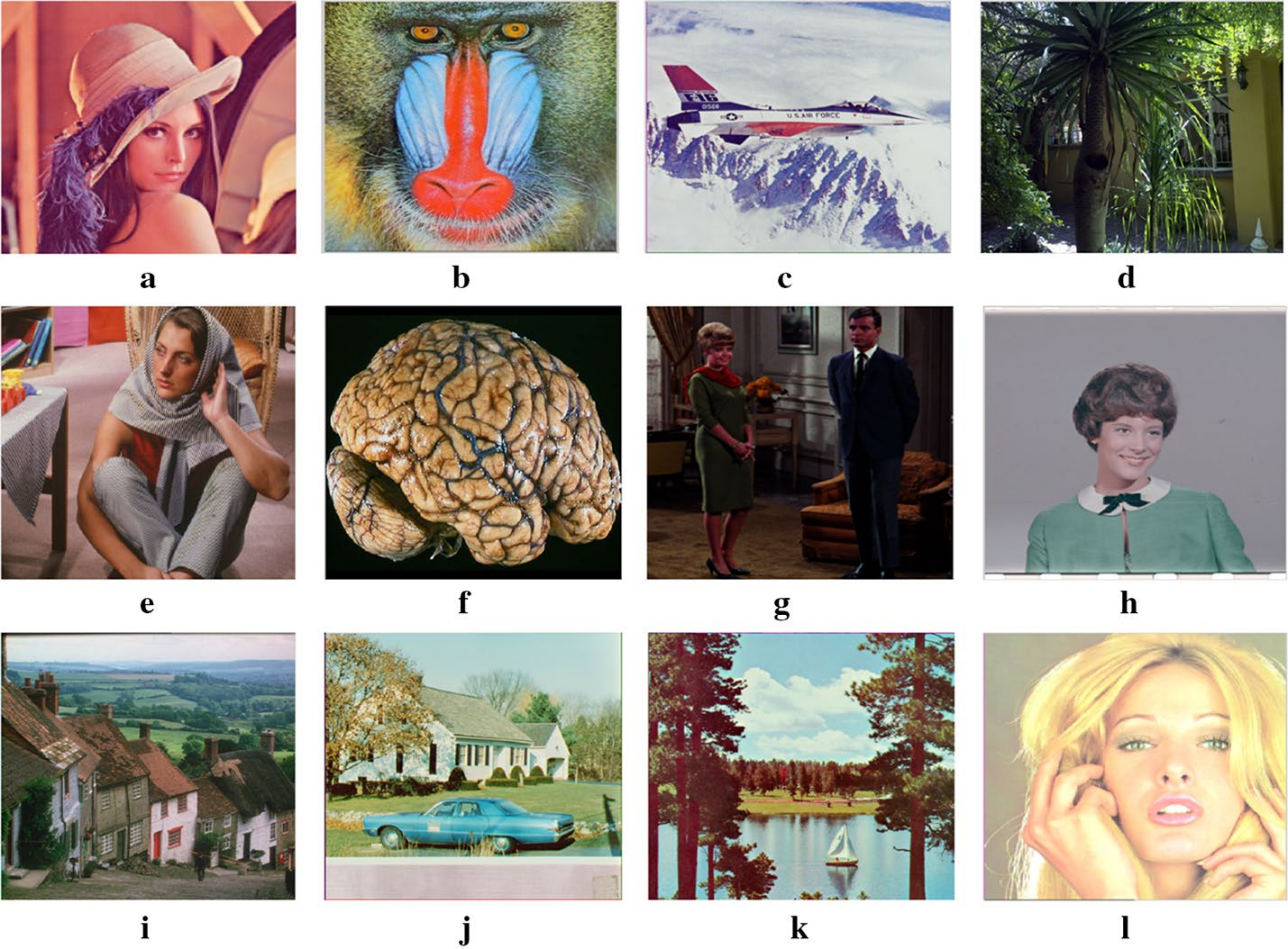
Test images: **a** Lena, **b** Mandrill, **c** Airplane, **d** Aptus, **e** Barbara, **f** Brain, **g** Couple, **h** Girl, **i** Gold hill, **j** House, **k** Lake and **l** Tiffany

The filters are evaluated in this section on a variety of RGB color images, which includes scientific images, biomedical images, photographic images, synthetic images and a group of images generally used in the color image processing literature, for a better comparison on the performance of the filters, by considering a 3 × 3 window size with *L*_1_ and *L*_2_ norms. Figure 4 shows the 12 representative images of Lena image, Mandrill, Airplane, Aptus, Barbara, Brain, Couple, Girl, Gold hill, House, Lake and Miramar.

### Noise model

An impulse noise model which is commonly used in the literature of filtering of color images is used in this work (Viero et al. 1994). Let *b* be the probability of corruption of the color image with the impulse noise. A color image has three vector components, where each component has a chance of being corrupted by the impulse noise with a respective corruption probability. Let *b*_*R*_, *b*_*G*_ and *b*_*B*_ be the probabilities of impulse noise corruption of the three components *R*, *G* and *B* respectively.

14 $$ \varvec{y} = \left\{ \begin{array}{l} x\,{\text{with}}\,{\text{probability}}\,\,1 - b \hfill \\ \left\{ {n_{R} ,x_{G} ,x_{B} } \right\}\,{\text{with}}\,{\text{probability}}\,\,b.b_{R} \hfill \\ \left\{ {x_{R} ,n_{G} ,x_{B} } \right\}\,{\text{with}}\,{\text{probability}}\,\,b.b_{G} \hfill \\ \left\{ {x_{R} ,\,x_{G} ,n_{B} } \right\}\,{\text{with}}\,{\text{probability}}\,\,b.b_{B} \hfill \\ \left\{ {n{}_{R},n_{G} ,\,n_{B} } \right\}\,{\text{with}}\,{\text{probability}}\,\,[1 - (b_{R} + b_{G} + b_{B} )].b \hfill \\ \end{array} \right. $$

$\varvec{x} = \left\{ {x_{R} ,x_{G} ,x_{B} } \right\}$ and $\varvec{y} = \left\{ {y_{R} ,y_{G} ,y_{B} } \right\}$ represent the original and the corrupted vector pixels respectively. And the impulse noise is represented by the random vector $n = \left\{ {n_{R} ,n_{G} ,n_{B} } \right\}$ which can be a vector of 0 or 255 or both. The images are induced with both fixed valued noise and random valued noise with noise ratio ranging from 10 to 90%. For the fixed valued noise, *η*_*k*_ can be either 0 or 225, whereas it can be any random value ranging from 0 to 255 for the random valued noise.

### Filter performance measurements

Execution time, mean absolute error (MAE) (Singh and Bora 2014), normalized color difference (NCD) (Singh and Bora 2014) and peak signal to noise ratio (PSNR) (Plataniotis and Venetsanopoulos 2000) are the performance measuring parameters which will be used to evaluate the filters in comparison. Color chromaticity preservation capability of a filter is measured with NCD. A filtered image is said to preserve its chromaticity if it is free from the shadowy effects whereas MAE represents the noise suppression and the signal–detail preservation capability. MAE is mathematically expressed as

15 $$ MAE = \frac{1}{3PQ}\sum\limits_{i = 1}^{P} {\sum\limits_{j = 1}^{Q} {\left[ \begin{array}{l} \left| {x(i,j)^{R} - x_{F} (i,j)^{R} } \right| + \left| {x(i,j)^{G} - x_{F} (i,j)^{G} } \right| \hfill \\ + \left| {x(i,j)^{B} - x_{F} (i,j)^{B} } \right| \hfill \\ \end{array} \right]} } $$

$\varvec{x}(i,j)$ and $\varvec{x}_{F} (i,j)\,$ are the original and the filtered image respectively. $\left\{ {x(i,j)^{R} ,x(i,j)^{G} ,x(i,j)^{B} } \right\}$ and $\left\{ {x_{F} (i,j)^{R} ,x_{F} (i,j)^{R} ,x_{F} (i,j)^{R} } \right\}$ are the red, green and blue components of the original image $\varvec{x}(i,j)$ and filtered image $\varvec{x}_{F} (i,j)$ respectively. It can be seen that with the help of the above equation a slight difference between the original and filtered image can be highlighted properly for better comparison between the filters. The NCD is defined in the $L\,u^{*} \,v^{*}$ color space by

16 $$ NCD = \frac{{\sum\nolimits_{i = 1}^{P} {\sum\nolimits_{j = 1}^{Q} {\left\{ {\left| {L(i,j) - L_{F} (i,j)\,} \right|^{2} + \left| {u\,\,(i,j) - u\,_{F} \,(i,j)\,} \right|^{2} + \left| {v\,(i,j) - v_{F} \,(i,j)\,} \right|^{2} } \right\}} }^{1/2} }}{{\sum\nolimits_{i = 1}^{P} {\sum\nolimits_{j = 1}^{Q} {\left\{ {\left| {L\,(i,j)\,} \right|^{2} + \left| {u\,(i,j)\,} \right|^{2} + \left| {v\,\,(i,j)\,} \right|^{2} } \right\}} }^{1/2} }} $$

where $\left\{ {L(i,j),u\,\,(i,j),v\,(i,j)} \right\}$ and $\left\{ {L_{F} (i,j),u\,_{F} \,(i,j),v_{F} \,(i,j)} \right\}$ are the respective values of the lightness and two chrominance components of the original image $\varvec{x}(i,j)$ and filtered image $\varvec{x}_{F} (i,j)$. And the signal content of the image is described by PSNR which is expressed as

17 $$ PSNR\, = \,10\,log\,_{10} \,\frac{{x^{2}_{max} }}{{\frac{1}{3PQ}\sum\nolimits_{i = 1}^{P} {\sum\nolimits_{j = 1}^{Q} {\left\| {x(i,j) - x_{F} (i,j)} \right\|} }^{2} }} $$

where $x_{max} = 2^{b} \, - 1$ is the maximum intensity for an image in a particular channel. 24 bits images of size 512 × 512 are considered in this work, where *b* = 8, is the number of bits in a pixel for the particular channel and *x*_*max*_ is equal to 255.

### Parameter selection

The exponential weight is found using Eq. 6 where the weight is controlled by the three parameters *α*, *η* and *β*. As seen from the weight equation, *α* and *η* are inversely proportional to the weight as seen in Eq. 8. Analyzing the simulation results on the various images, it is found that at the lower noise ratio, 10% and 20% as representative in this work, the PSNR attains its maximum value at lower value of ∝ = 0.4 and comparatively higher value of *η* = 0.6, supported by the fact that more weight is given by the first component $d(i)^{\alpha }$ in Eq. 8 to the pixels at lower noise ratio, during which most of the pixels in the window tends to be less impulse. Whereas the PSNR attains its maximum value at a relatively higher value of around *α* = 0.6 or 0.7 and comparatively lower value of *η* = 0.4 or 0.3 for higher noise ratio, during which less weight is necessary by the pixels in the window from the first component $d(i)^{\alpha }$ and instead more from the second component $l(i)^{\eta }$ is considered, since their probability to be impulse is more. Therefore $\alpha = 0.7\,,\,\eta \, = 0.3$ and $\alpha = 0.6,\,\,\eta \, = 0.4$ are considered for the highest noise ratio (80 and 90%) with relatively smaller value of *α* = 0.4 and higher value of *η* = 0.6 for lower noise ratio (10 and 20%) for fixed valued noise and random valued noise respectively (see Tables 1, 2). The above discussion concludes that the first component controlled the parameter *α* plays a bigger role as compared to the second component controlled by parameter *η* in Eq. 8. The values of *β* and $\partial$ are set as 1 and 0.5 respectively. Considering the Eqs. 4 and 5 which represents the expression for ACWVMF, the value of *ν* is considered as 3 so that *u* goes from 3 to 5, for which three different *x*_*CWVMF*_ is calculated with three different $w_{j} (u)$ respectively. The values of *T*_*L*_ and *T*_*H*_ in Eq. 3 are set as 0.000 and 0.111 respectively. The parameters and their respective values selection, for both fixed valued and random valued noise, for other filters in comparison are shown in Table 3.

**Table 3:** Filters in comparison (excluding the proposed filter), with their respective parameters’ values at which the filters are implemented

Filters	Parameters	Filters	Parameters
MMF		RCTDDF	*l* = 5, *T* = 40
VMF		FVMF	*α* = 0.5, *β* = 1
BVDF		FVDF	*α* = 2, *β* = 1
DDF	*μ* = 0.7	FOVMF	*α* = 0.5, *β* = 1, *l* = 4
CWVMF	*w* = 5	AVMMF	*l* = 5, *k* = 3
CWVDF	*w* = 3	SDM	*T*_*a*_ = 40, *T*_*b*_ = 70, *T*_*c*_ = 120, *T*_*d*_ = 160
CWDDF	*w* = 3	RSVMF	*β* = 0.15
ACWVMF	*w* = 3, *T* = 80	NCVMF	*T* = 32
ACWVDF	*w* = 5, *T* = 0.2	NCRVMF	*T* = 38
ACWDDF	*w* = 5, *T* = 47	NCVMF_2nc	*T* = 30
MCWVMF	*w* = 1.4	NCVMF_1nc	*T* = 20
PGF	*T* = 40	NCVMF_1c	*T* = 25
FPGF	*m* = 4, *T* = 30	SVMF_MEAN	*μ* = 1.544
AVMF	*T* = 35	SVMF_RANK	*μ* = 1.475
AVDF	$\partial$ = 1.57, *T* = 0.12	SVDF_MEAN	*μ* = 1.544
EVMF	*υ* = 0.6	SVDF_RANK	*μ* = 1.475
EVDF	*υ* = 0.2	SDDF_MEAN	*μ* = 1.544
EDDF	*υ* = 0.1	SDDF_RANK	*μ* = 1.475
NVMF	*T* = 90	ASVMF_MEAN	*δ* = 0.6
RCVMF	*l* = 5	ASVMF_RANK	*δ* = 0.65
RCVDF	*l* = 5	ASVDF_MEAN	*δ* = 1
RCDDF	*l* = 5	ASVDF_RANK	*δ* = 0.03
RCTVMF	*l* = 5, *T* = 40	ASDDF_MEAN	*δ* = 0.6
RCTVDF	*l* = 5, *T* = 50	ASDDF_RANK	*δ* = 0.5

### Comparison with other existing filters

**Fig. 5:**
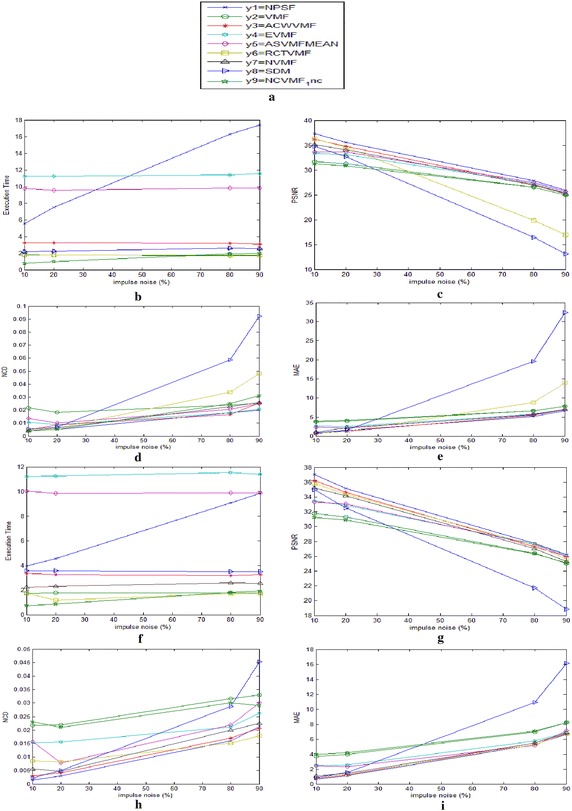
Comparison of some robust filters graphically: **a** legends depicting the filters in comparison and **b**–**e** shows execution time, PSNR, NCD and MAE comparison for fixed valued impulse noise; **f**–**i** shows execution time PSNR, NCD and MAE for random valued impulse noise

The performance comparison of the various filters is shown in Tables 4 and 5. Figure 5 shows the graphical comparison of the MASVMF_MEAN, VMF, ACWVMF, EVMF, ASVMF_MEAN, RCTVMF, NVMF, SDM and NCVMF_1nc. The PSNR which defines the signal content of the filtered image has been given more importance for comparing the performance. Filters like ACWVMF, ACWVDF, NVMF and PGF perform very well in lower noise ratio and also work efficiently in higher noise ratio. The RCTVMF and MCWVMF are very efficient in preserving the signal content in the lower noise ratio but perform inefficiently in the higher noise ratio. Whereas the EVMF and the vector sigma group of filters namely the ASDDF_RANK, ASVMF_MEAN, ASDDF_MEAN work efficiently in higher noise ratio although not very well in the lower noise ratio as compared to the case of ACWVMF and its allies mentioned above. But generally it can be clearly seen that the Adaptive switching filters like the Adaptive center weighted vector filters, Vector sigma filters, Adaptive vector filters and entropy based vector filters, first detect the noisy pixels using certain noise detection algorithm, before replacing the center pixel with the output of some vector filter and thus is able to give higher PSNR values than those of the non adaptive switching filters like the basic vector filters, fuzzy weighted filters etc. Therefore the adaptive switching filters are able to restore the original find details of the image better than the non-adaptive filters.

**Table 4:** Comparison of the filters for the Lena image induced with FVN at 10, 20, 80 and 90% based on NCD, PSNR, MAE and TIME

	FVN				FVN				FVN				FVN			
	.10				.20				.80				.90			
	NCD	PSNR	MAE	TIME	NCD	PSNR	MAE	TIME	NCD	PSNR	MAE	TIME	NCD	PSNR	MAE	TIME
VMF	0.021	31.753	3.720	1.76	0.018	31.289	3.937	1.76	0.023	26.574	6.64	1.70	0.025	24.964	7.79	1.73
VDF	0.719	30.029	4.477	11.53	0.020	29.219	5.120	11.60	0.042	18.658	15.30	11.96	0.071	13.839	29.46	11.86
DDF	0.020	31.696	3.771	26.55	0.020	31.219	3.993	26.79	0.025	26.417	6.71	27.35	0.022	24.943	7.93	27.15
ACWVMF	0.004	36.221	0.769	3.25	0.008	34.769	1.235	3.23	0.016	27.080	5.43	3.19	0.025	25.324	6.93	3.100
ACWVDF	0.015	35.024	0.974	41.45	0.016	32.957	1.606	40.96	0.037	18.705	13.19	38.53	0.063	14.164	26.05	38.15
ACWDDF	0.002	35.116	1.036	53.78	0.005	35.115	1.660	53.95	0.026	25.650	6.62	55.32	0.030	23.874	8.51	54.07
CWVMF	0.009	34.762	2.185	2.08	0.015	33.567	2.461	2.49	0.027	21.536	7.31	2.04	0.034	19.220	9.99	2.03
CWVDF	0.016	33.122	2.931	15.36	0.017	31.727	3.284	15.35	0.052	14.155	22.14	15.42	0.090	10.586	42.66	15.52
CWDDF	0.015	34.769	2.219	38.02	0.016	33.523	2.494	37.84	0.027	21.898	7.21	37.53	0.034	19.689	9.59	37.50
RCVMF	0.009	34.077	2.191	1.77	0.010	33.346	2.417	1.75	0.033	19.960	8.82	1.77	0.047	17.085	13.81	1.74
RCVDF	0.016	32.979	2.709	11.72	0.017	31.978	3.035	11.63	0.045	15.407	18.24	12.33	0.094	10.391	43.85	12.08
RCDDF	0.009	34.058	2.233	27.05	0.016	33.306	2.468	28.20	0.023	25.218	6.01	27.33	0.030	21.591	8.46	27.50
RCTVMF	0.004	36.257	0.791	1.80	0.006	34.576	1.262	1.78	0.033	19.828	8.85	1.77	0.048	16.965	14.00	1.77
RCTVDF	0.015	35.701	0.824	11.52	0.016	33.277	1.451	11.92	0.046	15.157	18.42	11.36	0.086	10.846	40.68	11.27
RCTDDF	0.004	36.306	0.794	26.63	0.016	34.648	1.261	26.52	0.032	19.917	8.84	27.12	0.046	17.159	13.67	26.79
EVMF	0.010	33.409	2.577	11.25	0.008	33.096	2.553	11.25	0.018	27.360	5.76	11.38	0.021	25.430	6.97	11.53
EVDF	0.019	30.509	4.359	16.99	0.019	29.795	4.503	23.86	0.041	17.724	15.55	25.97	0.075	12.575	33.03	24.26
EDDF	0.017	31.861	3.600	43.23	0.018	31.352	3.772	43.02	0.020	26.427	6.69	42.00	0.022	24.968	7.90	43.32
SVMF_RANK	0.005	35.424	1.427	1.71	0.006	33.943	1.598	1.71	0.027	22.316	6.89	1.69	0.037	19.205	10.29	1.69
SVDF_RANK	0.017	32.768	2.714	1.78	0.017	32.267	2.640	11.92	0.038	17.197	14.68	12.06	0.018	11.331	37.57	11.19
SDDF_RANK	0.006	34.982	1.664	26.98	0.006	34.296	1.730	26.84	0.025	23.739	6.37	27.02	0.031	20.807	8.89	26.62
SVMF_MEAN	0.010	32.731	2.702	10.21	0.010	32.475	2.503	10.10	0.022	26.691	5.87	9.42	0.026	24.347	7.40	9.51
SVDF_MEAN	0.017	32.507	2.765	25.49	0.017	31.817	2.571	23.80	0.043	15.955	16.71	26.96	0.086	11.016	39.54	22.49
SDDF_MEAN	0.016	34.855	1.760	30.60	0.016	34.414	1.718	28.98	0.024	24.071	6.31	24.95	0.032	20.864	9.17	24.95
ASVMF_RANK	0.016	33.739	2.166	10.88	0.016	33.459	2.001	10.81	0.022	27.127	5.66	10.68	0.025	24.968	7.11	10.70
ASVDF_RANK	0.013	30.095	4.735	12.37	0.020	29.245	5.124	12.37	0.041	18.898	15.07	13.50	0.071	13.824	29.35	13.40
ASDDF_RANK	0.010	33.191	2.457	37.85	0.016	33.027	2.236	38.35	0.017	27.033	5.86	36.12	0.025	25.369	7.12	35.02
ASVMF_MEAN	0.013	33.645	2.319	9.76	0.010	33.635	2.144	9.56	0.020	27.488	5.72	9.80	0.025	25.683	6.95	9.81
ASVDF_MEAN	0.015	34.481	1.323	22.73	0.017	30.532	1.817	22.52	0.075	13.958	21.95	22.85	0.110	11.785	33.06	23.17
ASDDF_MEAN	0.012	33.675	2.324	24.86	0.010	33.553	2.172	23.31	0.022	27.463	5.79	23.36	0.021	25.572	7.00	26.09
NCVMF	0.020	31.757	3.718	20.09	0.021	31.257	3.958	20.22	0.020	26.683	6.43	19.67	0.026	25.194	7.69	19.56
NCVMF_2C	0.021	31.632	3.753	19.30	0.022	31.184	3.969	19.44	0.023	26.527	6.53	19.29	0.026	24.901	7.77	19.76
NCVMF_1NC	0.022	31.250	3.969	18.91	0.019	30.858	4.173	19.32	0.024	26.524	6.62	19.35	0.024	24.921	7.82	19.34
NCVMF_1C	0.021	31.095	4.032	19.99	0.022	30.733	4.230	18.62	0.019	26.422	6.65	18.39	0.025	24.921	7.83	18.43
PGF	0.005	35.464	0.889	1.01	0.016	33.948	1.407	1.18	0.024	25.339	6.09	2.03	0.028	23.177	7.95	2.22
PGF_FAST	0.013	34.829	1.131	0.44	0.011	33.548	1.608	0.59	0.025	25.192	6.47	1.49	0.031	22.901	8.72	1.58
AVMF	0.006	34.899	1.016	1.84	0.005	33.727	1.498	1.82	0.016	26.856	5.66	1.78	0.021	25.109	7.24	1.76
AVDF	0.015	35.203	0.762	15.32	0.016	32.340	1.475	15.14	0.039	17.492	14.42	15.23	0.071	12.810	30.52	15.01
MCWVMF	0.005	35.522	1.354	1.76	0.006	33.912	1.541	1.71	0.040	18.254	10.71	1.73	0.058	15.762	16.46	1.69
NVMF	0.003	35.170	1.068	2.18	0.006	34.093	1.499	2.23	0.022	27.008	5.54	2.58	0.025	25.194	7.03	2.53
SDM	0.005	34.771	0.872	3.86	0.006	32.649	1.561	3.71	0.058	16.417	19.55	3.63	0.092	13.124	32.34	3.70
MMF	0.013	33.061	3.211	0.23	0.011	31.916	3.455	0.24	0.035	18.556	9.94	0.24	0.044	16.803	12.97	0.24
RSVMF	0.014	33.522	2.428	1.75	0.012	33.149	2.410	1.75	0.022	26.689	5.76	1.70	0.026	24.368	7.20	1.73
FVMF	0.018	32.107	3.697	6.94	0.020	31.633	3.890	6.99	0.027	26.341	7.05	6.96	0.025	24.599	8.75	6.94
FVDF	0.020	31.827	4.217	14.11	0.022	30.559	4.863	14.34	0.074	16.888	28.37	14.13	0.105	13.950	41.29	14.04
FOVMF	0.013	31.303	4.047	7.68	0.013	30.643	4.333	7.64	0.025	23.921	8.95	7.66	0.037	21.640	12.15	7.76
AVMMF	0.017	32.764	2.908	5.03	0.016	32.011	3.207	5.29	0.027	24.770	7.70	5.20	0.034	22.053	10.29	5.17
NCRVMF	0.020	31.729	3.727	2.65	0.019	31.290	3.945	2.96	0.020	26.761	6.49	2.85	0.025	25.200	7.66	2.74
NPSF	0.004	37.315	0.668	5.563	0.005	35.541	1.148	7.475	0.018	27.866	5.154	16.28	0.020	25.931	6.65	17.37

**Table 5:** Comparison of the filters for the Lena image induced with RVN at 10, 20, 80 and 90% based on NCD, PSNR, MAE and TIME

	RVN				RVN				RVN				RVN			
	.10				.20				.80				.90			
	NCD	PSNR	MAE	TIME	NCD	PSNR	MAE	TIME	NCD	PSNR	MAE	TIME	NCD	PSNR	MAE	TIME
VMF	0.021	31.743	3.73	1.74	0.021	31.283	3.96	1.77	0.031	26.388	6.95	1.76	0.032	25.046	8.25	1.76
VDF	0.013	29.979	4.77	11.59	0.014	29.091	5.18	11.85	0.045	19.612	14.94	11.53	0.066	16.277	23.56	11.43
DDF	0.021	31.709	3.78	27.66	0.021	31.239	4.01	26.59	0.029	26.344	7.01	27.50	0.032	24.826	8.54	27.72
ACWVMF	0.003	36.217	0.75	3.34	0.004	34.641	1.23	3.25	0.017	27.292	5.48	3.18	0.020	25.986	6.78	3.23
ACWVDF	0.002	34.758	0.09	40.13	0.007	32.846	1.59	40.60	0.033	20.535	11.80	39.98	0.053	17.373	18.77	40.90
ACWDDF	0.011	35.562	0.87	55.46	0.003	34.127	1.35	55.46	0.018	26.337	6.11	56.18	0.022	25.147	7.76	56.64
CWVMF	0.009	34.864	2.17	2.08	0.015	33.855	2.43	2.08	0.026	25.471	6.03	2.07	0.024	23.837	7.46	2.07
CWVDF	0.008	32.993	2.94	41.02	0.010	31.669	3.30	40.25	0.034	19.104	13.52	40.62	0.047	16.932	19.01	40.65
CWDDF	0.016	34.820	2.20	37.56	0.009	33.976	2.46	37.37	0.023	25.855	6.00	37.66	0.025	23.940	7.48	37.03
RCVMF	0.010	34.037	2.19	1.75	0.007	33.328	2.42	1.75	0.019	27.637	5.55	1.77	0.021	25.851	6.71	1.75
RCVDF	0.007	33.054	2.70	11.52	0.009	31.870	3.04	11.32	0.029	21.441	10.49	11.79	0.041	18.337	15.88	11.72
RCDDF	0.010	33.997	2.23	26.93	0.011	33.448	2.45	26.59	0.016	28.016	5.48	27.10	0.023	25.983	6.78	26.74
RCTVMF	0.008	35.794	0.91	1.72	0.008	34.389	1.35	1.17	0.015	27.500	5.29	1.70	0.017	25.624	6.70	1.71
RCTVDF	0.003	34.666	1.08	11.39	0.004	32.902	1.65	11.38	0.028	21.401	10.15	11.94	0.041	18.174	16.08	11.37
RCTDDF	0.008	35.700	0.93	28.44	0.008	34.518	1.35	28.36	0.016	27.896	5.20	27.13	0.020	25.823	6.72	27.01
EVMF	0.015	33.482	2.49	11.21	0.015	32.888	2.54	11.24	0.021	27.680	5.76	11.53	0.026	26.132	6.93	11.39
EVDF	0.013	30.287	4.06	25.20	0.018	29.417	4.96	24.64	0.044	19.872	14.25	24.95	0.059	17.052	21.30	24.57
EDDF	0.020	31.685	3.76	44.31	0.012	31.287	3.97	44.59	0.032	26.292	7.14	43.72	0.336	25.092	8.37	43.71
SVMF_RANK	0.003	35.140	1.49	1.73	0.004	34.164	1.66	1.74	0.019	26.442	5.49	1.68	0.021	24.365	6.99	1.70
SVDF_RANK	0.008	32.537	2.86	12.18	0.009	32.010	2.85	11.95	0.030	21.168	11.00	11.48	0.045	17.932	17.25	11.62
SDDF_RANK	0.004	34.882	1.72	27.01	0.006	34.094	1.83	27.10	0.014	27.086	5.49	25.72	0.021	25.180	6.83	25.12
SVMF_MEAN	0.007	32.549	2.86	10.21	0.011	32.370	2.73	10.43	0.020	27.097	6.07	9.50	0.023	25.917	7.12	9.49
SVDF_MEAN	0.090	32.085	2.93	25.30	0.014	31.482	2.80	23.78	0.031	20.524	11.58	22.13	0.049	17.229	18.70	21.85
SDDF_MEAN	0.009	34.724	1.83	30.97	0.009	34.076	1.84	30.34	0.015	27.190	5.53	37.48	0.021	25.274	7.00	39.75
ASVMF_RANK	0.006	33.459	2.30	11.54	0.006	33.124	2.18	11.62	0.021	27.586	5.66	11.29	0.022	26.111	6.80	11.26
ASVDF_RANK	0.013	30.032	4.74	12.24	0.014	29.258	5.13	12.33	0.046	19.775	14.81	12.76	0.061	17.171	21.30	12.81
ASDDF_RANK	0.010	32.880	2.63	39.15	0.010	32.776	2.44	39.72	0.024	27.467	5.90	38.93	0.021	25.764	7.19	38.72
ASVMF_MEAN	0.015	33.374	2.45	10.01	0.007	33.083	2.35	9.84	0.021	27.562	5.16	9.88	0.030	25.970	7.15	9.86
ASVDF_MEAN	0.003	34.651	1.34	23.27	0.006	33.291	1.78	23.24	0.026	21.171	9.57	22.60	0.035	18.701	14.05	22.64
ASDDF_MEAN	0.006	33.356	2.47	24.68	0.011	32.978	2.38	24.54	0.023	27.468	5.97	24.12	0.027	25.998	7.15	24.89
NCVMF	0.020	31.739	3.73	20.07	0.022	31.286	3.96	19.18	0.029	26.421	6.97	19.31	0.031	25.287	8.08	19.26
NCVMF_2C	0.022	31.690	3.75	19.25	0.022	31.209	3.98	19.36	0.028	26.418	6.97	19.73	0.036	24.964	8.25	19.25
NCVMF_1NC	0.023	31.232	3.98	19.49	0.021	30.870	4.19	19.55	0.030	26.342	7.06	19.88	0.029	25.104	8.24	19.46
NCVMF_1C	0.020	31.088	4.04	19.23	0.021	30.698	4.26	19.53	0.030	26.494	6.99	20.21	0.036	25.191	8.26	20.03
PGF	0.003	35.301	0.89	0.99	0.004	33.657	1.43	1.11	0.018	26.321	6.03	1.88	0.023	24.874	7.57	2.01
PGF_FAST	0.013	34.851	1.13	0.42	0.010	33.465	1.62	0.57	0.020	25.673	6.60	1.36	0.025	24.013	8.50	1.51
AVMF	0.002	34.895	1.00	1.83	0.012	33.586	1.49	1.83	0.017	26.824	5.88	1.79	0.023	25.369	7.38	1.73
AVDF	0.013	29.988	4.77	16.13	0.014	29.077	5.19	15.32	0.048	19.264	15.59	15.64	0.057	17.245	21.48	15.57
MCWVMF	0.005	35.393	1.40	1.88	0.004	34.342	1.58	2.03	0.018	26.059	5.55	1.94	0.021	24.014	7.08	1.90
NVMF	0.005	35.219	1.05	2.22	0.004	34.192	1.46	2.28	0.019	27.055	5.49	2.57	0.022	25.300	6.84	2.56
SDM	0.002	34.978	0.84	3.55	0.005	32.543	1.56	3.57	0.028	21.714	10.94	3.48	0.045	18.873	16.13	3.50
MMF	0.013	33.207	3.22	0.23	0.014	32.390	3.44	0.24	0.028	22.850	7.88	0.24	0.035	21.139	9.79	0.25
RSVMF	0.014	33.310	2.54	1.76	0.016	33.010	2.56	1.78	0.022	27.203	5.71	1.75	0.021	26.001	6.95	1.76
FVMF	0.019	32.193	3.68	7.01	0.021	31.732	3.88	7.00	0.032	26.179	7.25	7.98	0.038	24.838	9.04	7.94
FVDF	0.022	31.983	4.16	14.05	0.024	31.170	4.59	14.21	0.060	23.213	12.70	14.10	0.067	21.336	15.95	14.19
FOVMF	0.021	31.484	4.01	7.45	0.022	30.863	4.28	7.49	0.027	24.885	8.38	7.51	0.032	23.124	10.58	.4382
AVMMF	0.009	32.789	2.90	5.14	0.012	32.191	3.17	5.36	0.030	25.889	7.33	6.56	0.026	24.358	8.98	6.78
NCRVMF	0.020	31.794	3.71	2.68	0.022	31.301	3.96	2.69	0.031	26.520	6.91	2.85	0.033	25.101	8.19	2.50
NPSF	0.0016	37.063	0.655	3.95	0.003	35.161	1.147	4.56	0.016	27.748	5.28	9.06	0.0212	26.237	6.65	9.85

**Fig. 6:**
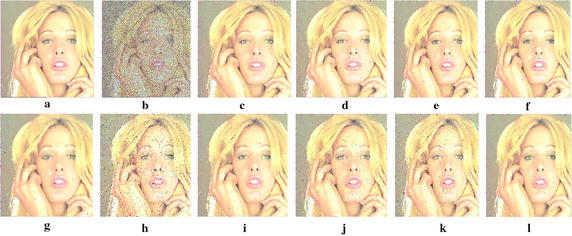
Filtering results for the TIFFANY image corrupted with 90% noise: **a** original, **b** 90% noisy, **c** ASVMF_MEAN, **d** ASVMF_RANK, **e** ASDDF_MEAN, **f** ASDDF_RANK, **g** VMF, **h** MCWVMF, **i** NVMF, **j** RSVMF, **k** RCTVMF and **l** MASVMF

**Fig. 7:**
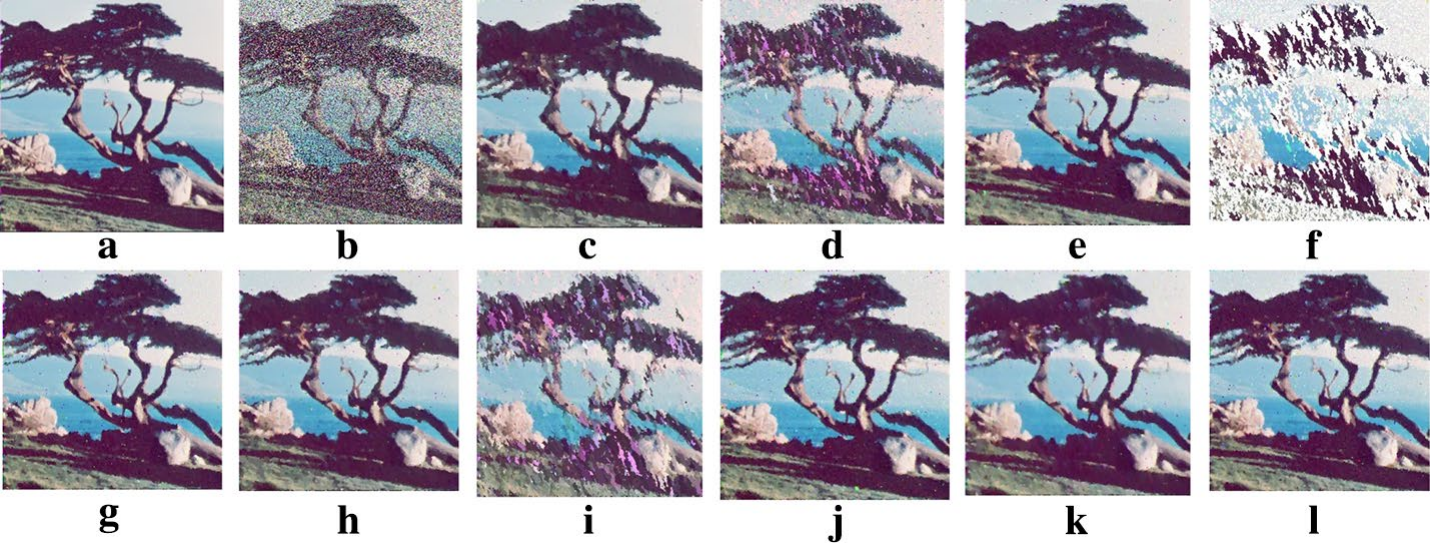
Filtering results for the TREE image corrupted with 90% noise: **a** original, **b** 90% noisy, **c** ACWDDF, **d** ACWVDF, **e** ACWVMF, **f** AVDF, **g** AVMF, **h** EDDF, **i** EVDF, **j** EVMF, **k** FVMF and **l** MASVMF

After working on various numbers of images it can be seen that the proposed filter, Noise percentage based switching filter maintains a good PSNR value at lower noise ratio by outperforming robust filters like ACWVMF, ACWVDF, etc. and also definitely overtakes even the most robust filters, which are very efficient in the higher noise ratios, like ACWVMF, EVMF and some vector sigma filters. This is supported by the experimental results shown in the Tables 4 and 5, where the results of some efficient filters in consideration are christened. Figures 6 and 7 show the original images, corrupted images with 90% of fixed valued and random valued impulse noise and filtered images of Tiffany and Tree respectively. The filtered images of the proposed filter are shown in image *l* of the Figs. 6 and 7, depicting high signal content and detail preservation. But seen precisely the filtered images are little blurred since the noisy pixels are replaced by the output of the average of the exponentially weighted filter. And also the proposed filter can be further improved in terms of preservation of chromaticity by furthering lowering the value of NCD.

## Conclusions

The proposed filter is able to outperform the other robust filters, in maintaining the signal content and preserving the fine details of the image corrupted with fixed valued and random valued impulse noise at the lower noise ratios as well as at the higher noise ratios. And the filter also maintains the chromaticity of the filtered image at a lower value of NCD and MAE as compared to some of the very efficient filters in literature. The weighted average output of the filter does not belong to the vectors in the window because of which the filter can be extended further for Gaussian noise and mixed Gaussian and impulse noise removal. Other than this, an unwanted commonly occurring issue called smoothing can be still further minimized. Future work will be in introducing a directional distance factor in the exponential weight function so that the chromaticity maintenance of the image is improved further.
